# Ammonia Recovery from Animal Manure via Hollow Fibre Membrane Contactors: Impact of Filtration Pre-Treatment and Organic Foulants on Mass Transfer and Performance

**DOI:** 10.3390/membranes16010015

**Published:** 2025-12-31

**Authors:** Niloufar Azizi, Shaun Connolly, Dominika Krol, Eoin Syron

**Affiliations:** 1 School of Chemical and Bioprocess Engineering, University College Dublin, D04 V1W8 Dublin, Ireland; 2 Environment, Soils and Land Use Department, Teagasc, Johnstown Castle, Y35 TC97 Wexford, Ireland

**Keywords:** filtration, mass transfer coefficient, membrane fouling, membrane contactor, ammonia recovery, animal manure

## Abstract

Ammonia (NH_3_) recovery from animal manure offers both environmental and economic benefits by reducing nitrogen emissions and producing valuable fertilisers. Hollow fibre membrane contactors (HFMCs) are a promising technology for this purpose, yet their performance is strongly influenced by the complex composition of manure. In this study, the effects of solids concentration and organic foulants concentration on the mass transfer coefficients governing NH_3_ recovery were systematically investigated. Total suspended solids (TSS) were reduced through graded filtration, and protein concentrations in the ammonium solutions were quantified to assess their role in limiting mass transfer. Results showed that TSS concentration primarily affected the shell-side film resistance. After extensive filtration, residual proteins attached to the membrane surface induced partial wetting, thereby reducing the effective membrane mass transfer coefficient. Using a penalty function approach, it was possible to separately describe TSS- and protein-related resistances, enabling improved prediction of effective model coefficients under real world conditions. These findings highlight the dual importance of solid–liquid separation and protein management in optimising HFMC operation for NH_3_ recovery and provide a framework for up-scaling the technology in agricultural nutrient management systems.

## 1. Introduction

Organic waste is responsible for 70% of anthropogenic ammonia (NH_3_) emissions [1], with agriculture contributing over 81% of global NH_3_ emissions [2]. The intensive production of livestock generates large quantities of livestock manure, which produces greenhouse gases and NH_3_, while inadequate treatment and poor management leads to increased emissions into the atmosphere and pollution of water bodies [3]. Animal manure contains high levels of organic matter, nutrients, and residual emerging contaminants, threatening both human health and the environment if improperly managed. Total Ammoniacal Nitrogen (TAN) concentrations in animal manure range from 500 to 6000 mg L^−1^ [4]. Currently, most nitrogen fertilisers are produced via the Haber–Bosch process. This process is known to be energy-intensive and environmentally damaging. Therefore, nitrogen recovery from waste streams can help address the global need for fertiliser and reduce emissions to the atmosphere as well as the nitrogen load on wastewater treatment plants and downstream effluent-receiving waters [5].

Nitrogen recovery from agricultural manure is possible through methods such as NH_3_ stripping, struvite precipitation, and membrane separation [6]. NH_3_ stripping is the most advanced technology within the European Union (EU), as noted by Rizzioli et al. [6]. It removes TAN from manure by transferring it to the gas phase and then capturing it in an acid solution, typically sulfuric acid (H_2_SO_4_), to produce ammonium sulphate (AS) [7,8]. NH_3_ stripping is regarded as an effective technique, capable of achieving nitrogen removal rates of up to 90–95% in just a few hours [9]. This process can be carried out in packed-bed towers to maximise the surface area for mass transfer, necessitating the addition of heat or chemical additives and a high removal of solids in the slurry to prevent clogging of the stripping column [10]. Although it is implemented in waste treatment plants [11], the application of NH_3_ stripping on livestock farms remains quite limited. Struvite precipitation enables the recovery of phosphorus and NH_3_ as an ammonium magnesium phosphate hexahydrate ((NH_4_)Mg(PO_4_)·6H_2_O). This method is mainly utilised in the municipal wastewater sector, especially for treating anaerobic sewage sludge digestates, but its use within agriculture is relatively rare, as outlined by Rizzioli et al. [6].

The separation of the digestate and manure into a liquid and solid fraction is recommended for reducing animal waste volumes and the costs associated with transportation [12]. However, solid–liquid separation does not guarantee a high recovery of nutrients still available in the liquid fraction.

The membrane separation process has shown potential for NH_3_ recovery from wastewater. Membrane contactors have generated considerable interest for controlling NH_3_ in industrial applications, as well as carbon dioxide and fluid deoxidation [13]. Membrane contactors have potential to be a suitable technology for the removal and recovery of NH_3_ in wastewater and agricultural manure because the operation of the membrane contactor is relatively simple. During liquid-phase operation, ammonia from the manure solution diffuses across the hydrophobic membrane into an acidic receiving solution (typically H_2_SO_4_), resulting in the direct formation of liquid fertilisers such as ammonium sulphate or ammonium sodium nitrate [14,15]. Hydrophobic membrane contactors have the advantage of operating at ambient temperature, and acids in the receiving solution can react with NH_3_, maintaining a high NH_3_ concentration gradient across the membrane, which is the driving force of transmembrane transport. This method can achieve the selective separation of NH_3_ with minimal energy and chemical input, which can effectively reduce the cost of the recovery process. Among different types of membrane module configurations, Hollow Fibre Membrane Contactors (HFMCs) have the advantages of high packing density and large surface area per volume over other membrane modules, which results in high efficiency and relative cost savings of widespread application in NH_3_ recovery from wastewater [16].

Membrane fouling remains a persistent challenge in HFMCs treating complex feeds such as animal manure. Therefore, solid–liquid separation is a critical pretreatment step in NH_3_ recovery from agricultural manure, as it enhances the performance and longevity of downstream processes. Solids can cause operational issues like foaming, clogging, and fouling and may carry reactive compounds that interfere with recovery efficiency or product purity [17,18]. Separating the solid fraction not only facilitates the recovery of valuable resources such as phosphorus and organic matter but also helps reduce solids and organic load in the liquid phase. Nonetheless, pathogens and dissolved organics can persist in the liquid fraction, which may interfere with downstream processes. Therefore, appropriate pretreatment, such as solid–liquid separation or filtration, is essential to enhance process efficiency, protect equipment, and improve the overall sustainability of ammonia recovery systems.

Analysing animal slurry particle sizes is challenging due to a lack of standardised methods. While sieving is common, particles under 1 μm (colloids) are hard to remove due to their unique movement. Different slurries have varying particle distributions; for instance, pig slurry typically has a higher proportion of smaller particles than cattle slurry [19]. Microbial degradation processes, including anaerobic digestion and natural aging during storage, progressively break down undigested material, leading to a higher proportion of smaller particles in older manure. Understanding the nutrient content within these particle fractions is crucial, as a large portion of the nitrogen (N) and phosphorus (P) in cattle slurry is found in specific size ranges. However, data on nutrient content in fine particles within the micrometre range (below 15 µm, 8 µm, and 1.2 µm) are still limited, while submicron particles were not considered in this study [4].

Fouling in HFMCs is known to be caused by organic macromolecules, especially proteins, which adsorb onto or into the membrane, forming gel layers that increase membrane-side resistance and lower the local mass-transfer coefficient (k_m_) [20,21]. Another source of fouling, Total Suspended Solids (TSS), promote the development of particulate cake or roughness layers, raising shell-side resistance and reducing shell-side mass transfer coefficient (ks) [22]. Most traditional mass transfer correlations do not explicitly consider these foulants. Conventional Sherwood-style or concentration-polarisation models for km and ks are typically based on flow conditions and physical properties, and seldom include foulant concentration as a variable. Few studies have proposed corrections for concentration-dependent or TSS-modulated mass-transfer coefficients in HFMCs. Key findings from AlSawaftah et al. [23] include the governing roles of feed composition, hydrodynamics, and membrane properties in pressure-driven fouling, and that mitigation relies on pretreatment, optimized strategies, and predictive models for pore blocking, cake formation, and concentration polarization. Yang et al. [24] further demonstrated that extending these models with attachment coefficients, and applying computational fluid dynamics, Monte Carlo simulations, and machine learning, offers deeper insights. Although much work targets desalination and water treatment, these fouling mechanisms are equally relevant to gas–liquid membrane contactors.

In line with these theoretical insights, experimental work has shown that additional resistances beyond those predicted by clean hydrodynamic correlations must be considered. For example, Mavroudi et al. [25] demonstrated that CO_2_ absorption in water using HFMCs is initially controlled by the liquid phase, but over time, partial pore wetting introduces a reversible membrane resistance. Even limited liquid penetration (<13% of the pore length) accounted for 21–53% of the total resistance, underscoring the importance of accounting for non-idealities in Resistance-In-Series (RIS) models.

Building on these theoretical and experimental foundations, the present work adapts the RIS concept to NH_3_ recovery from animal manure. Here, the focus is not on time-dependent wetting but on composition-dependent resistances arising from suspended solids and organic foulants, particularly proteins. This study examines a HFMC system for NH_3_ recovery from animal manure. A saturating fouling-resistance model was developed and used to quantify the effect of solid particle and protein concentrations on NH_3_ mass transfer. These findings allow for the validated model to be used for the design and scaling up of HFMC systems.

## 2. Materials and Methods

### 2.1. Ammonium Solution

In this study, both an ammonium solution and cattle manure were used to investigate the factors effecting mass transfer in a HFCM.

All chemicals and reagents utilised in this study were of analytical grade unless otherwise specified and were used as received without further purification. Specifically, ammonium chloride (NH_4_Cl) and gelatin from cold water fish skin (powder, MW: 60 kDa) (Sigma-Aldrich, St. Louis, MO, USA), sodium hydroxide (NaOH) (SAFC, St. Louis, MO, USA), sulfuric acid (95% H_2_SO_4_) (Fisher Chemical, Loughborough, UK) and hydrochloric acid (≥37% HCl, Product No. 30721-2.5L) (Honeywell Fluka, Charlotte, NC, USA). Probumin^®^ Bovine Serum Albumin Vaccine Grade (Merck Life Science Limited, Darmstat, Germany) (powder, MW: 66 kDa) was also used.

To mimic proteins in manure the synthetic solutions were supplemented with Gelatin and Bovine Serum Albumin (BSA) [26,27]. The selection of a protein concentration range of 0.4 to 2 g/L for both BSA and gelatin in synthetic solutions were based on a literature review of protein concentrations in the liquid fraction of pre-treated animal manures [12,28]. While raw manure contains high total protein, a significant portion is associated with solid particles. The chosen range of 0.4, 0.8, 1.2, 1.6, and 2 g/L aims to realistically simulate the concentration of proteinaceous foulants present in finely filtered manure. BSA was selected as a representative globular protein commonly used in fouling studies, while gelatin was chosen to explore the impact of more fibrous or gel-forming protein characteristics, both of which can be found in the complex organic matrix of manure filtrates.

### 2.2. Manure Characterisation

The cattle manure used in this study was collected from UCD Research Lyons farm Co Kildare, Ireland. The cattle manure was analysed, and the results are shown in Table 1. The following parameters were analysed for the raw manure samples, conducted under the following protocols in house: total solids (TS, method: SM-2540-B) [29], total suspended solids (TSS, method: SM-2540-D) [30], and total volatile solids (TVS, methods: SM-2540-E) [31].

### 2.3. Pre-Treatment: Manure Filtration

**Figure 1:**
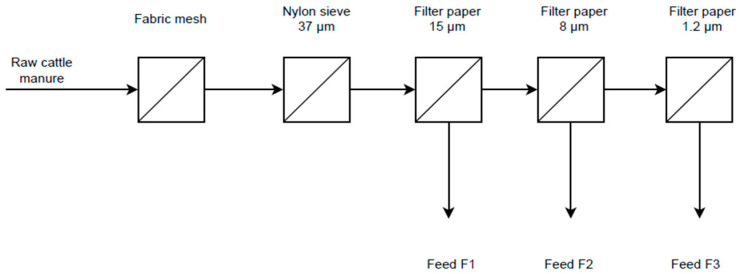
Schematic of the steps taken for pretreatment of the cow manure for solid–liquid filtration prior to ammonia recovery.

The manure was initially passed through a stainless-steel electric-driven tomato strainer (VEVOR, Suzhou, China) as a screw press (mesh size 1.5 mm) and was then subsequently filtered using progressively smaller filter sizes. The filters used had pore sizes of 37 μm, 15 µm, 8 µm, and 1.2 µm. Figure 1 illustrates the filtration procedure, where F1, F2, and F3 represent feed streams with filtration sizes of 15 µm, 8 µm, and 1.2 µm, respectively. Filtrate, which has passed through the 1.2 µm filters, has primarily soluble and colloidal organic matter, including proteins remaining. The filtered manure sample was diluted (1:100) following filtration, and the ammonium concentrations were adjusted back to the original manure sample concentration by adding NH_4_Cl. Protein concentration in the filtered manure was determined following filtration through a 1.2 µm membrane.

### 2.4. HFMC System and Operation

**Figure 2:**
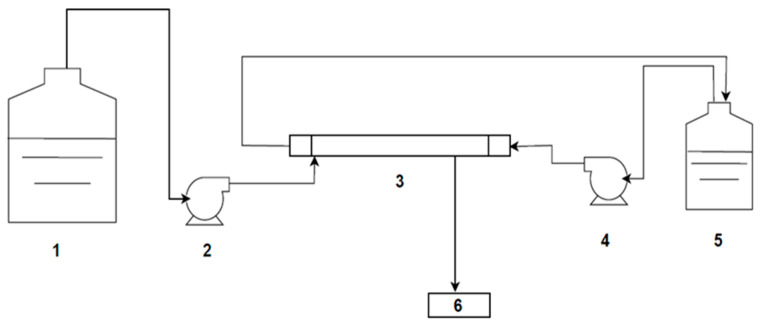
Open loop process, with number representation as follows: 1. Ammonium solution/filtered manure reservoir, 2, 4. pumps, 3. Hollow Fibre Membrane Contactor, 5. absorption solution (sulfuric acid) reservoir, and 6. stripped solution/filtered manure.

A schematic representation of the experimental setup used for NH_3_ removal is shown in Figure 2. It consisted of an HFMC (1 × 5.5 Liqui-Cel Membrane Contactor X-50 PP fibre) (3M Company, St Paul, MN, USA). The HFMC module worked in an open loop and a counter-current mode. Silicone flexible tubes connected the two tanks, containing feed and acid stripping solutions, which were pumped to the lumen and shell sides, respectively.

This lab system was prepared following the methodology proposed previously by Zhang et al. [32] as an open-loop system. Table 2 provides the details of the 1 × 5.5 Liqui-Cel Membrane.

The stripping solution was pumped and circulated into the lumen side by using a peristaltic dosing pump (SEKO), while the feed was pumped into the shell side of the membrane module using a Masterflex L/S Compact Drive peristaltic pump (Cole-Parmer, Vernon Hills, IL, USA). These types of pumps allow an easily adjustable flow rate. A 1 mol L^−1^ sodium hydroxide solution was used to adjust the pH of the feed to 11, and 0.25 mol L^−1^ sulfuric acid solution was used as the stripping solution. All the tests, including process time, sample collection, and flow rate adjustment, ran for 3.0 h. Experiments were carried out at various feed flowrates according to the operating conditions outlined in Table 3.

### 2.5. Analytical Methods

TAN concentration throughout the recovery process was monitored using an Orion™ AquaMate™ 9500 Portable Multiparameter Meter (ThermoFischer Scientific, Waltham, MA, USA) coupled with an Orion™ 9512HPBNWP Ammonia Electrode (ThermoFischer Scientific, Waltham, MA, USA). This setup allowed for accurate and real-time measurement of NH_3_ levels following each flowrate adjustment. To ensure optimal conditions for NH_3_ stripping, the pH of all feed solutions was adjusted to 11 using an Orion™ Star A111 pH Benchtop Meter (ThermoFischer Scientific, Waltham, MA, USA). Before starting the recovery process, the concentrations of proteins, gelatin, and BSA in the feed solutions were determined using a NanoDrop One/OneC Microvolume UV-Vis Spectrophotometer (ThermoFischer Scientific, Waltham, MA, USA).

Only the clearest filtered manure (F3) could be measured for protein because F1 and F2 were too turbid and dark, causing light scattering, unstable baselines, and instrument errors. Therefore, protein values for F1 and F2 were not directly measurable and were instead treated as estimated “protein-equivalent foulant” inputs (based on the F1 value).

To assess membrane fouling and its reversibility, a standardised test was conducted using an ammonium solution both before and after each experiment. This allowed for evaluation of the membrane’s performance decline and potential for recovery. Following each set of experiments utilising manure and protein-containing samples, the membrane contactor underwent a thorough cleaning procedure based on the methodology outlined in Zarebska et al. [27]. This established cleaning protocol aimed to mitigate fouling and restore membrane performance for subsequent experimental runs. Table 4 provides details related to the size of the filter, dilution, and TSS and protein concentrations of experiments for this study.

### 2.6. Theoretical Model Development

Overall mass transfer efficiency (*K_ov_*) for NH_3_ transport was determined by Equation (1).

(1) $$K_{ov}=\frac{Q}{A}ln\frac{C_{0}}{C}$$

where *Q* is the feed (ammonium solution/the manure) flowrate (m^3^ s^−1^), *A* is the membrane area (m^2^), and *C*_0_ is N-NH_3_ concentration in influent, and *C* is N-NH_3_ concentration in effluent.

A RIS model was developed to describe NH_3_ transfer through the HFMC. The overall mass transfer coefficient (*K_ov_*) was expressed by Equation (2) [33].

(2) $$\frac{1}{K_{ov}}=\frac{1}{k_{m}}+\frac{1}{k_{s}}+\frac{1}{k_{l}}$$

where *k_s_*, *k_m_*, and *k_l_* represent the shell-side, membrane, and lumen-side mass transfer coefficients, respectively. Since the lumen side contained a strong acid solution, the concentration of NH_3_ in the bulk liquid was assumed to be negligible. Therefore, the lumen resistance term (1/*k_l_*) was neglected [34].

(3) $$\frac{1}{K_{ov}}=\frac{1}{k_{m}}+\frac{1}{k_{s}}$$

The shell-side mass transfer coefficient (*k_s_*) can be predicted by Sherwood correlations in the general form [35]:

(4) $$Sh=\;\frac{k_{s}d_{e}}{D_{A,w}}=Af(\Phi ){\left(\frac{d_{e}}{l}\right)}^{B}{Re}^{C}{Sc}^{D}$$

where *D_A,w_* is the diffusion of NH_3_ in water (m^2^ s^−1^), *d_e_* and *l* are the hydraulic diameter, and the length of fibre respectively, *A*, *B*, *C*, and *D* are constants from the correlation of the experimental data and *f*(*Φ*) is a function of packing densities. *Re* and *Sc* are Reynolds and Schmit numbers, respectively. There are more than 30 Sherwood correlations in the literature to predict the mass transfer coefficient on the shell side [35,36]. The correlation varied with module type, Reynolds number, packing fraction, and feed flow rate. Table 5 shows the five chosen Sherwood correlations which align with the experimental process conditions.

The membrane mass transfer coefficient can be calculated by the following equation [34]:

(10) $$k_{m}=\;\frac{D_{A,m}H\;\varepsilon }{\tau \delta }$$

where *D_A,m_*, *H*, *ε*, *τ*, and *δ* are the diffusion coefficient of NH_3_ in membrane pores, dimensionless Henry’s law constant, porosity, tortuosity, and wall thickness of hollow fibre, respectively.

Diffusion of NH_3_ in membrane pores can be calculated by Equation (11).

(11) $$\frac{1}{D_{A,m}}=\frac{1}{D_{kn}}+\frac{1}{D_{A,air}}$$

where *D_kn_* is the Knudsen diffusion diffusivity (m^2^ s^−1^) and can be calculated by Equation (12). *D_A,air_* is NH_3_ diffusivity in air (m^2^ s^−1^) and equals 1.89 × 10^−5^ m^2^ s^−1^.

(12) $$D_{kn}=\frac{d_{p}}{3}\sqrt{\frac{8RT}{\pi M}}$$

where *M*, *R*, and *d_p_* are the molecular weight of NH_3_ (g mol^−1^), universal gas constant (J mol^−1^ K^−1^), and pores diameter (m), respectively.

### 2.7. Incorporation of Fouling

The impact of suspended solids and organic foulants on mass transfer was incorporated through empirical additional resistance functions. On the shell side, TSS concentration was used as a correction factor in Equation (13), and in the membrane, protein concentration was applied as an indicator of pore fouling and adsorption in Equation (14).

(13) $$k_{s,pred}=k_{s,clean}f(TSS)$$

(14) $$k_{m,pred}=k_{m}f(C_{P})$$

where *f*(*TSS*) represents the reduction in shell-side mass transfer caused by suspended solids and *f*(*C_p_*) is an empirically determined reduction factor caused by protein concentration.

The effective membrane coefficient was also estimated by back-calculation from experimental data:

(15) $$\frac{1}{k_{m,eff,exp}}=\frac{1}{K_{ov,exp}}-\frac{1}{k_{s,pred}}$$

The experimentally determined *k_m,eff,exp_* values were compared with the model-predicted *k_m,pred_* obtained from the proposed penalty function. Comparison of the two methods (penalty-based vs. back-calculated) provided independent estimates of the membrane contribution to the total resistance.

### 2.8. Model Calibration and Validation

Experimental NH_3_ concentrations were determined by measuring inlet and outlet concentrations of the manure side and absorbent side using an ion-selective electrode.

The accuracy of model predictions was evaluated by comparison with experimental data. Model performance was assessed using Root Mean Square Error (RMSE) and Mean Absolute Percentage Error (MAPE). Additionally, a 1:1 parity plot was used to visually assess the agreement between predicted and observed coefficients. All data analysis and parameter estimation were performed using the Solver add-in in Microsoft Excel (Microsoft Corporation, Redmond, WA, USA).

## 3. Results and Discussion

The RIS model was used to investigate the NH_3_ mass transfer in the open-loop HFMC. Based on the model, to get the overall mass transfer coefficient (*k_ov_*), mass transfer in the shell side (*k_s_*) and membrane pore (*k_m_*) should be predicted. The overall mass-transfer coefficient was interpreted with RIS on a liquid-phase basis by neglecting lumen-side resistance in Equation (4).

### 3.1. Experimental Validation of Shell-Side Mass Transfer Correlations

**Figure 3:**
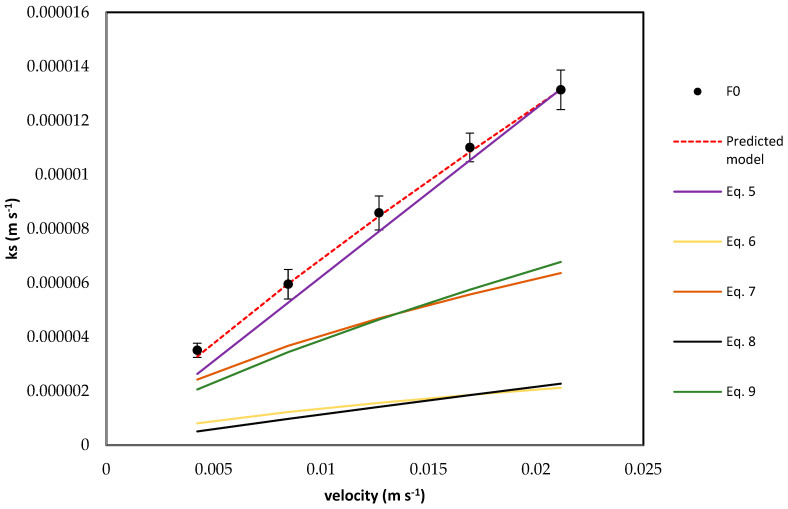
Comparison of experimentally measured shell-side mass transfer coefficient with chosen empirical Sherwood correlations in Table 4.

The five empirical correlations presented in Table 4 were compared with the experimental data. A new correlation for *k_s_* prediction given by Equation (16) was developed and validated by experimental *k_s_* values. Shell-side coefficients were regressed on the synthetic dataset using Equation (16). Figure 3 shows that the experimental and predicted *k_s_* (R^2^ = 0.998) increase with increasing feed velocities over the range 0.0042–0.0212 m/s.

(16) $${Sh}_{clean}=12.42\left(\frac{d_{e}}{L}\right){Re}^{0.86}{Sc}^{0.33}$$

Figure 3 reveals clear differences between correlations. Correlations that (i) use superficial rather than interstitial velocity, (ii) adopt the different characteristic length (hydraulic diameter instead of fibre OD) (Equations (6) and (8)), or (iii) assume different packing or geometry tend to over-predict at low velocity or under-predict at high velocity. The baseline predicted Sherwood number from the clean solution curve aligns with the ammonium solution across the entire velocity range, whereas several literature correlations deviate in slope and intercept. This figure supports the decision to recalibrate the clean Sherwood correlation on the module and to treat matrix effects as penalties layered on that baseline, rather than reusing off-the-shelf correlations.

### 3.2. Effect of Solids Concentration on Mass Transfer

The effect of *TSS* on mass transfer can be captured with a single multiplicative penalty on the Sherwood correlation *Sh_pred_* = *f*(*TSS*)*Sh_clean_*. In Equation (17), *α*, *β*, and *γ* are constants (Table 6), and the *TSS* scale was chosen as 110 mg L^−1^ to stabilise the fit.

(17) $$f\left(TSS\right)=\frac{1}{{\left(1+\alpha {\left(\frac{TSS}{{TSS}_{scale}}\right)}^{\beta }\right)}^{\gamma }}$$

The fixed vertical gaps between *TSS* levels at a given velocity is captured by the single *f*(*TSS*) multiplier, confirming that filtration acts on the shell film, not on the hydrodynamic slope [42].

**Figure 4:**
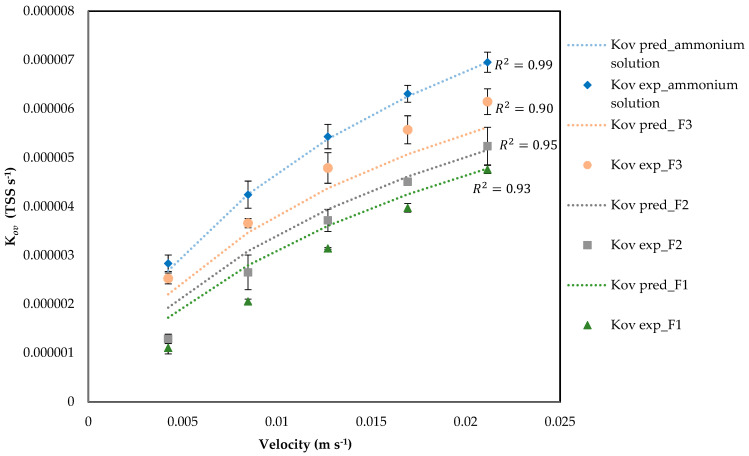
Comparison of predicted shell-side mass transfer, including *TSS* penalty for different filtration sizes. Dashed lines indicate *K_ov_* calculated by predicted *k_s_*.

Using the proposed correlation Equation (16), the effect of solids concentration was investigated. Figure 4 plots the Sherwood and Reynolds number for experiments carried out with increasing *TSS* concentrations. It can be observed that increasing TSS concentrations lead to a reduction in the Sh number for a given Re number. Fitted linear correlations between Sh and Re show that F1, F2, and F3 are almost parallel with the slope of approx. 0.21, while the ammonium solution has a slope of 0.27. This shows that the predictions correlate very well with experimental results. When the parameters Sh vs. Re are plotted, the data points corresponding to the lowest Re of 2.47 were omitted from the Figure 4 as they deviated from the general trend and obscured the linear relationship.

**Figure 5:**
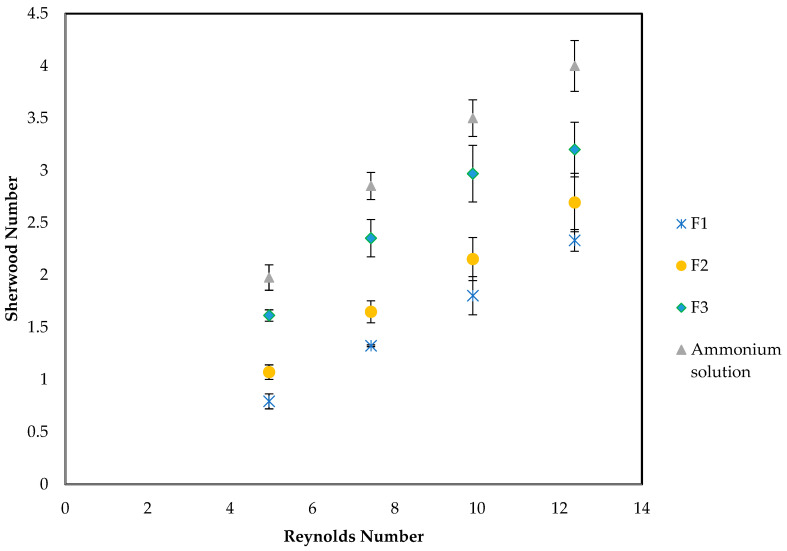
Plot of Sherwood and Reynolds numbers from experiments carried out at different *TSS* concentrations.

Plotting the change in mass transfer coefficient with changing velocity for the four samples shows that the mass transfer coefficient decreases significantly with an increase in solids concentration (Figure 5). The increase of *K_ov_* with velocity in Figure 4 reflects film thinning. This indicates external boundary-layer effects (viscosity/turbulence damping and thin cake/gel), rather than changes in hydrodynamic scaling [43]. Figure 5 through the corresponding datasets, indicates that suspended solids primarily act by increasing shell-side resistance rather than altering the flow exponent [44].

### 3.3. Effect of Proteins on the Membrane Coefficient

The mass transfer rate of ammonium was measured for solutions with a BSA and gelatine concentrations of 0.4–2 g L^−1^, and using Equation (15) the effective membrane coefficient for each run was determined.

**Figure 6:**
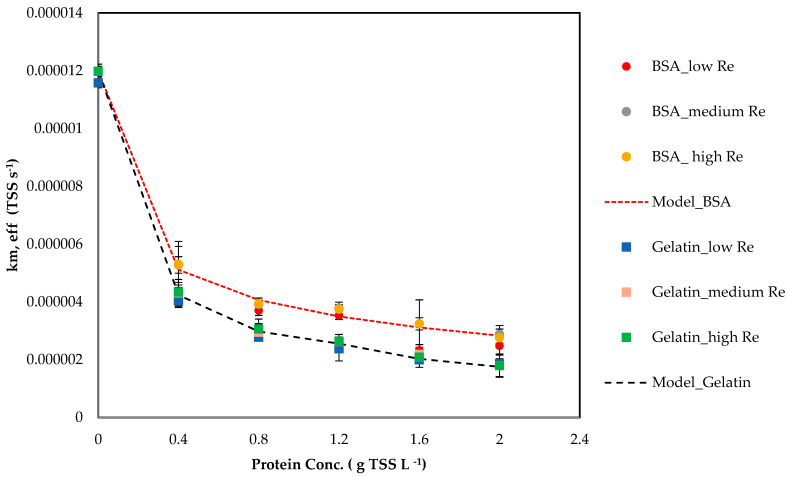
*k_m,eff_* vs. protein concentration. Points: back-calculated *k_m,eff,exp_* (BSA, gelatin using Equation (15)). Dashed line: model *k_m,pred_* (Equations (21) and (22)).

Figure 6 shows *k_m,eff,exp_* decreasing with increasing protein concentrations, for both BSA and gelatin. No impact of a change in *k_m_* was observed with the change in velocity but there was a slight difference in *k_m_* between BSA and gelatin. These results are consistent with adsorption/partial wetting occurring and increasing membrane resistance [45].

A compact three-parameter model captures the decline in *k_m_* for both proteins with two different parameter sets, despite the differences in molecular weight and size for each protein. However, we recommend defining single parameters of *α_p_*, *β_p_*, and *γ_p_* for proteins in the cattle manure when assessing membrane mass transfer coefficients.

The proteins were modelled with a three-parameter penalty on *k_m_*:

(18) $$f(Cp)=\frac{1}{{\left(1+\alpha_{p}{\left(\frac{C_{p}}{C_{p\;scale}}\right)}^{\beta_{p}}\right)}^{\gamma_{p}}},$$

By using *C_p,scale_* = 0.4 g/L and *α_p_*, *β_p_*, and *γ_p_*, from Table 7, fitted to BSA and gelatin protein data, which reproduced the data, the *k_m,eff,exp_* trend (Figure 4, dashed line).

**Figure 7:**
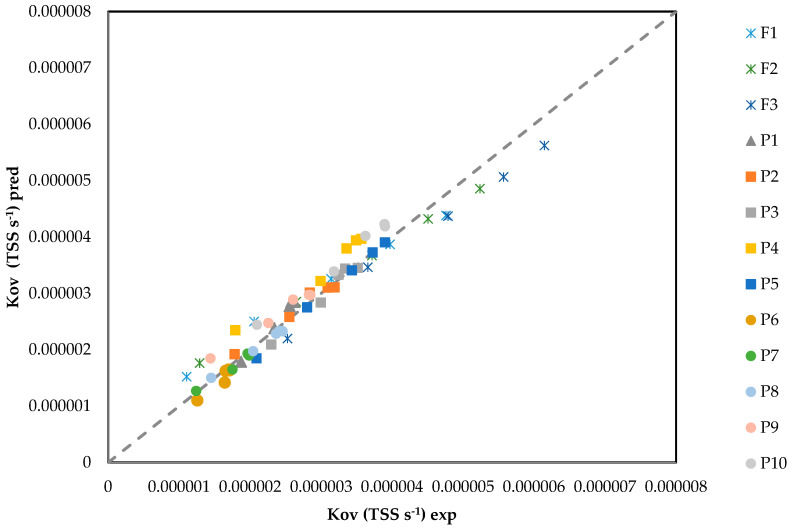
*K_ov,pred_* (Equation (19)) vs. *K_ov,exp_* (Equation (1)) for all experiments conducted.

Figure 7 shows *K_ov-pred_* vs. *K_ov-exp_* for both protein concentration evaluation and TSS concentration evaluation. Equations (19)–(22) show how the *K_ov,pred_*_,_ *k_s,pred_*, and *k_m,pred_* for BSA and gelatin were calculated, respectively. It was not possible to measure protein concentrations in F1 and F2 by the NanoDrop method due to particle interference. Protein concentration in F3 was measured at 0.018 mg L^−1^ and, using this value, protein in F1 and F2 was estimated, using a proportionality to the measured TSS by Equation (18). Incorporating these *C_p_* values into *k_m,eff_*(*C_p_*) changed *K_ov_* by <1% across velocities; thus, Figure 5 differences are governed by the TSS (film-side) effect.

It is necessary to mention that for the calculation of *K_ov,pred_* for TSS evaluation, *k_m_*(*C_p_*) and *k_s_(TSS)* were considered. This figure shows good agreement across sizes (MAPE = [7.46]%).

(19) $$\frac{1}{K_{ov,pred}}=\frac{1}{k_{s,pred}}+\frac{1}{k_{m,pred}},$$

(20) $$k_{s,pred}=k_{s,clean}\;\frac{1}{{\left(1+0.28{\left(\frac{TSS}{110}\right)}^{1.06}\right)}^{0.77}}\;,$$

(21) $$k_{m,pred}BSA=\frac{k_{m}}{{\left(1+0.53{\left(\frac{C_{p}}{0.4}\right)}^{0.38}\right)}^{2.3}},$$

(22) $$k_{m,pred}\;gelatin=\frac{k_{m0}}{{\left(1+0.76{\left(\frac{C_{p}}{0.4}\right)}^{0.58}\right)}^{2}},$$

By utilizing the *k_s,pred_* in conjunction with *k_m,eff,pred_*(*C_p_*), precise predictions of the *K_ov_* values across various conditions, including clean samples, filtrate, and intentional protein additions, can be achieved. Figure 7 demonstrates a near unit slope with a small intercept, indicating random residuals without bias concerning Re, *TSS*, or *C_p_*. This supports the simplicity of employing one hydrodynamic slope along with two compact penalties applied consistently on a liquid-phase basis.

### 3.4. Model Prediction

**Figure 8:**
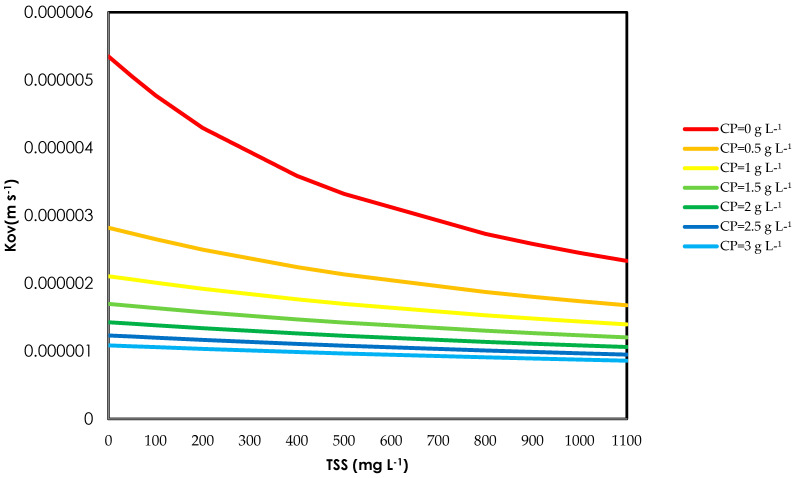
Model prediction for mass transfer coefficient *K_ov,pred_* (Equation (19)) for increasing TSS concentration at varying protein concentration levels.

Using Equations (20) and (22), *K_ov_* was predicted for *TSS* = 0–1100 mg L^−1^ and protein = 0–3 g/L (Figure 8). The model shows that *K_ov_* decreases with increasing *TSS* for all protein levels, and that dissolved protein has an even stronger penalising effect than *TSS*. For example, at negligible *TSS*, raising protein from 0 to 3 g/L reduces *K_ov_* by about 80%, whereas increasing TSS from 0 to 1100 mg L^−1^ at *C_p_* = 0 g/L leads to a two- to three-fold drop. The model indicated that the dissolved protein concentration governs *K_ov_*, especially at higher *C_p_*, at lower *C_p_* both TSS and *C_p_* influences *K_ov_*, but by increasing *C_p_* the system becomes increasingly controlled by organic foulants rather than suspended solids. These predictions emphasise that effective pretreatment must target both solids and dissolved organics to sustain high mass transfer coefficients during NH_3_ recovery from manure-derived feeds.

The RIS model was calibrated and evaluated using the manure dataset within the experimentally tested concentration range; therefore, predictions outside this range are considered extrapolations and carry higher uncertainty. To further support the protein-related resistance term without turbidity-related measurement limitations, additional controlled tests using gelatine as a surrogate proteinaceous foulant over 0–5 g L^−1^ were performed. (Supplementary Information). Although gelatine does not fully represent the complexity of manure soluble organics, the additional data support the trend of increasing protein concentrations leading to increased membrane-related resistance. Accordingly, additional experiments at higher protein concentrations and using real manure at elevated concentrations would be beneficial in validating the model. Future work should validate the RIS model using independent manure datasets (including additional TSS/protein levels and different manure types) and extended-duration experiments to quantify time-dependent fouling and refine parameter robustness [13,46,47].

### 3.5. Practical Implications

Filtration of the manure to remove *TSS* lowers the shell-side resistance and makes *K_ov_* higher, but the improvements get smaller compared to the ammonium solution when *TSS* is already low because there are fewer solids left to remove. The single integrated model also gives useful design predictions for different matrices without the need to adjust hydrodynamics.

Figure 4 and Figure 5 show that post-filtration, there is an increase in *K_ov_*, but once TSS is already very low, the process is mainly limited by the membrane itself. Further filtration has little effect unless proteins are also reduced.

At a *TSS* concentration of 315 mg L^−1^, the main resistance comes from the shell liquid film, so raising the velocity is very effective at reducing the overall mass transfer resistance. When proteins are present, the membrane itself becomes the main barrier, and increasing velocity shows little improvement. In these cases, the results indicate that focusing on the membrane properties to increase the rate of mass transfer, for example, through cleaning, applying anti-wetting coatings, or adjusting maintenance practices. This is in keeping with the findings of Goh et al. [48] and Gruskevica and Mezule [49], who showed that protein-rich organic foulants can form a dense layer on the membrane surface under wastewater treatment conditions. This layer becomes the dominant resistance to mass transfer, leading to severe flux decline and wetting, and they highlighted that appropriate chemical or enzymatic cleaning is needed to recover the intrinsic membrane mass-transfer coefficient.

Residual proteins in filtrates, even at μg L^−1^ levels, can be accounted for via the term *k_m,eff_*(*C_p_*). For the ranges observed here, these residual proteins change *K_ov_* by well below 1%. Therefore, design choices for operation of the membrane contactor should be driven by reducing k_s_ rather than k_m_.

A limitation of this study is that residual *C_p_* in the manure samples was measured for only one condition F1 and this value was used to estimate results for other cases. Conducting targeted experiments at various TSS levels without any associated proteins would improve accuracy and reduce uncertainty. Additionally, long-term effects such as progressive wetting and irreversible adsorption were not considered. Incorporating a time-dependent effective membrane mass transfer coefficient could facilitate monitoring of these changes. Furthermore, although the clean Sherwood slope was consistently applied across the module, it is necessary to evaluate the sensitivity of results to packing and bundle variations before generalizing to other systems.

Although this study captures how solid and dissolved foulants affect RIS mass transfer during short HFMC runs, no evaluation of how the system behaves over longer periods of continuous operation or repeated use were conducted. In real world applications, when membrane contactors treat real waste-derived streams, fouling can build up gradually: particles and organics can accumulate on the membrane surface, organics may adsorb over time, and partial wetting can develop. As these changes progress, the main resistance to mass transfer can change, and the removal efficiency may decline compared with the initial performance. Long-term studies and reviews consistently identify fouling and wetting as key constraints in membrane contactor-based ammonia recovery and emphasise the need for extended operation and cleaning-cycle evaluation when assessing practical applicability and scale-up [13,46,50].

The results of this study show that protein-induced *k_m_* reduction can be described using protein molecular descriptors, rather than just an empirical factor. Specifically, *α_p_*, *β_p_*, and *γ_p_* are expected to scale with molecular size and diffusivity, which can be represented by molecular weight and hydrodynamic radius, as well as with net charge at the operating pH, isoelectric point (pI), and hydrophobicity. However, the current dataset includes only two proteins, limiting robust assessment of relationships between *α_p_*, *β_p_*, *γ_p_*, and these descriptors. Therefore, expanding the protein panel to cover a wider range of molecular weights, isoelectric points, and hydrophobicity is recommended to develop unified, descriptor-based functions for *α_p_*, *β_p_*, and *γ_p_*.

This study used cattle manure only, so applying the results to other manure types should be done cautiously. Because slurry properties (especially particle size distribution and organic composition) vary with animal source and storage, the effectiveness of pretreatment, the rate of fouling/wetting, and the relative importance of shell-side vs. membrane-related resistances may change across different matrices [19,27,51,52]. Therefore, while the RIS framework should remain conceptually useful, its parameters and dominant resistances should be re-validated when switching feedstocks. As future work, it is recommended to extend this study to other manure types (e.g., pig slurry) under comparable operating conditions to test generalisability and refine model parameters accordingly.

## 4. Conclusions

This study investigated the performance of HFMCs for NH_3_ recovery from animal manure, with a particular focus on the role of pretreatment and organic foulants in determining the overall mass transfer coefficient. The results showed that the highest values of *K_ov_* were obtained with the clean synthetic solution, while the filtered manure exhibited reduced performance; manure with high *TSS* concentrations yielded the lowest values. These results show that suspended solids mainly increase shell-side resistance, whereas soluble organic fractions, such as proteins, reduce the membrane-coefficient through adsorption. The fixed reduction of *K_ov_* between samples across all velocities further confirmed that *TSS* acts mainly as an additional resistance, without altering the hydrodynamic slope. Through RIS analysis, the distinct contributions of solids and proteins were quantified. *TSS* effects were best captured by a correction to the shell-side coefficient, while protein effects were expressed through a concentration-dependent multiplier in the membrane-side coefficient. This dual resistance framework offers a new mechanistic understanding of how filtration pretreatment alters the limiting step of mass transfer in HFMC systems. Importantly, the NanoDrop analysis confirmed that even after 1.2 µm filtration, residual protein fractions remained in the manure, explaining the reduced *k_m_* relative to the clean solution. These findings demonstrate that effective pretreatment strategies for manure-based NH_3_ recovery should prioritise suspended solids removal over soluble organic foulants.

## Figures and Tables

**Table 1:** Cattle manure characteristics from a dairy farm, collected directly from a tank under a slatted shed.

Parameters	Unit	Value
Total Solids	%	10.07 ± 0.05
Total Volatile Solids	%	8.19 ± 0.32
Total Nitrogen	mg L^−1^	1490 ± 15
N-NH_4_^+^	mg L^−1^	741 ± 7
P-PO_4_	mg L^−1^	158 ± 1
K^+^	mg L^−1^	3200 ± 32
pH	-	7.40

**Table 2:** Information on 1 × 5.5 MiniModule *.

Module Type	1 × 5.5 MiniModule^TM^
Number of Fibres, N	2300
Porous polypropylene hollow fibre type	X-50
Effective module Length, L_eff_ (cm)	11.5
Membrane surface area (cm^2^)	0.1828
Inner radius, Ri (cm)	0.011
Outer radius, Ro (cm)	0.015
Pore diameter, dp (cm)	4 × 10^−6^
Porosity, ε	0.4

* Module data sheet; hollow fibre end spotted with epoxy in the tube sheet in the MiniModule^TM^.

**Table 3:** Experimental operating conditions for the operation of the membrane contactor for both ammonium solution and filtered manure.

Operation	Unit	Conditions
Flowrate	L/h	5, 10, 15, 20, 25
Acid flowrate	L/h	15
Acid Concentration	mol L^−1^	0.25
pH of Ammonium Solution/Filtered manure	-	11
N-NH_4_^+^	mg L^−1^	780 ± 20
BSA/Gelatin in Ammonium Solution	g L^−1^	0.4, 0.8, 1.2, 1.6, and 2
Temperature	°C	20

**Table 4:** Solutions used in experiments, labels related to the filtration, all solutions had a TAN concentration of 780 ± 20 mg L^−1^.

Name	Filter	Dilution	TSS (mg L^−1^)	Protein (g L^−1^)
F0	None	Ammonium solution	0	0
F1	20 µm	Cattle manure × 100	315	Not measured
F2	8 µm	Cattle manure × 100	220	Not measured
F3	1.2 µm	Cattle manure × 100	110	0.018
P1	None	BSA	0	2
P2	None	BSA	0	1.6
P3	None	BSA	0	1.2
P4	None	BSA	0	0.8
P5	None	BSA	0	0.4
P6	None	Gelatin	0	2
P7	None	Gelatin	0	1.6
P8	None	Gelatin	0	1.2
P9	None	Gelatin	0	0.8
P10	None	Gelatin	0	0.4

**Table 5:** Empirical Sherwood correlations used in this study.

Authors	Correlations	Equation Number	References
Dahuron and Cussler	$Sh=8.8(Re\frac{d_{e}}{L}){Sc}^{0.33}$	(5)	[37]
Prasad and Sirkar	$Sh=5.8(\frac{d_{e}(1-\Phi )}{L}){Re}^{0.6}{Sc}^{0.33}$	(6)	[38]
Basu and Sirkar	$Sh=17.4(\frac{d_{e}(1-\Phi )}{L}){Re}^{0.6}{Sc}^{0.33}$	(7)	[39]
Yang and Cussler	$Sh=1.25{\left(Re\frac{d_{e}}{L}\right)}^{0.6}{Sc}^{0.33}$	(8)	[40]
Viegas and Crespo	$Sh=8.71(\frac{d_{e}}{L}){Re}^{0.74}{Sc}^{0.33}$	(9)	[41]

**Table 6:** Constants values in Equation (18).

Constants	Values
*α*	0.28
*β*	1.06
*γ*	0.77

**Table 7:** Constant values used in Equation (19).

Constants	BSA	Gelatin
*α_p_*	0.53	0.76
*β_p_*	0.38	0.58
*γ_p_*	2.3	2

## Data Availability

The raw data supporting the conclusions of this article will be made available by the authors on request.

## Supplementary Materials

The following supporting information can be downloaded at: https://www.mdpi.com/article/10.3390/membranes16010015/s1, Figure S1: Overall mass transfer rate for experiments using clean water with increasing gelatine concentrations of 0.4–5 g/L for increasing liquid velocities.

## Nomenclature

**Variables**	
*K*	Mass Transfer Coefficient (m·s^−1^)
*CR*	Ammonia capture rate (g·h^−1^)
*a*	Membrane area (m^2^)
*Q*	Feed flowrate (m^3^ s^−1^)
*C*	Concentration of ammonia (mg L^−1^)
*L*	Length of fibre (m)
*d*	Diameter (m)
*Φ*	Packing fraction
*Re*	Reynolds number
*Sc*	Schmit number
*Sh*	Sherwood number
*τ*	Tortuosity
*δ*	Wall of fibre thickness (m)
*H*	Henry’s law Constant (dimensionless)
*ε*	Porosity
*R*	Universal gas constant (J mol^−1^ K^−1^)
*T*	Temperature (K)
*M*	Molecular weight (g mol^−1^)
*N*	Number of Fibres
*v*	Velocity (m s^−1^)
*α*	Constant
*β*	Constant
*γ*	Constant
**Subscripts**	
*K_ov_*	Overall Mass Transfer Coefficient (m s^−1^)
*k_m_*	Membrane Mass Transfer Coefficient (m s^−1^)
*k_s_*	Shell-side Mass Transfer Coefficient (m s^−1^)
*C*_0_	Initial concentration of ammonia in influent (mg L^−1^)
*C_P_*	Initial concentration of protein (g L^−1^)
*k_L_*	Lumen-side Mass Transfer Coefficient (m s^−1^)
*d_e_*	Hydrodynamic Diameter (m)
*d_p_*	Pore Diameter (m)
*D_A,W_*	Ammonia Diffusivity in water (m^2^ s^−1^)
*D_A,air_*	Ammonia Diffusivity in air (m^2^ s^−1^)
*D_kn_*	Knudsen Diffusivity (m^2^ s^−1^)
*D_A,m_*	Ammonia Diffusivity in membrane (m^2^ s^−1^)
*k_S,PRED_*	Shell-side Mass Transfer Coefficient predicted by Equation (13) (m s^−1^)
*k_S,CLEAN_*	Shell-side Mass Transfer Coefficient predicted by ammonium solution (m s^−1^)
*k_m,pred_*	Membrane Mass Transfer Coefficient predicted by Equation (14) (m s^−1^)
*k_m,eff,exp_*	Effective Membrane Mass Transfer Coefficient (m s^−1^)
*K_ov,exp_*	Overall Mass Transfer Coefficient calculated by Equation (1) (m s^−1^)
*L_eff_*	Effective module Length (cm)
*TSS_scale_*	Initial TSS considered in the experiment (mg L^−1^)
*C_P scale_*	Initial protein concentration considered in the experiment (mg L^−1^)
*k_m_*_0_	Back-calculated Membrane Mass Transfer Coefficient from experiments (m s^−1^)
*α_p_*	Protein Constant for k_m_ calculation
*β_p_*	Protein Constant for k_m_ calculation
*γ_p_*	Protein Constant for k_m_ calculation
**Acronyms**	
TAN	Total Ammoniacal Nitrogen
AS	Ammonium Sulphate
EU	European Union
HFMC	Hollow Fibre Membrane Contactor
*TSS*	Total Suspended Solid
BSA	Bovine Serum
RMSE	Root Mean Square Error
MAPE	Mean Absolute Percentage Error
RIS	Resistance In Series
OD	Outer Diameter of fibre
µm	Micrometre
