# Supplementary material for: Ammonia Recovery from Animal Manure via Hollow Fibre Membrane Contactors: Impact of Filtration Pre-Treatment and Organic Foulants on Mass Transfer and Performance
Source: Membranes (Basel). 2025 Dec 31;16(1):15. doi: 10.3390/membranes16010015 (PMC12843982; doi:10.3390/membranes16010015)
Supplement: Supplementary file 1 [file membranes-16-00015-s001.zip › membranes-4052128-supplementary.pdf]

SUPPLIMENTARY INFORMATION

The effect of hydrodynamic conditions on overall mass transfer was evaluated using gelatine as a controlled proteinaceous foulant. Figure S1 presents the variation of K_{OV} with liquid velocity for gelatine concentrations spanning 0–5 g L^{-1}. Across all cases, increasing velocity increased K_{OV}, consistent with reduced boundary-layer thickness and improved convective renewal at higher shear.

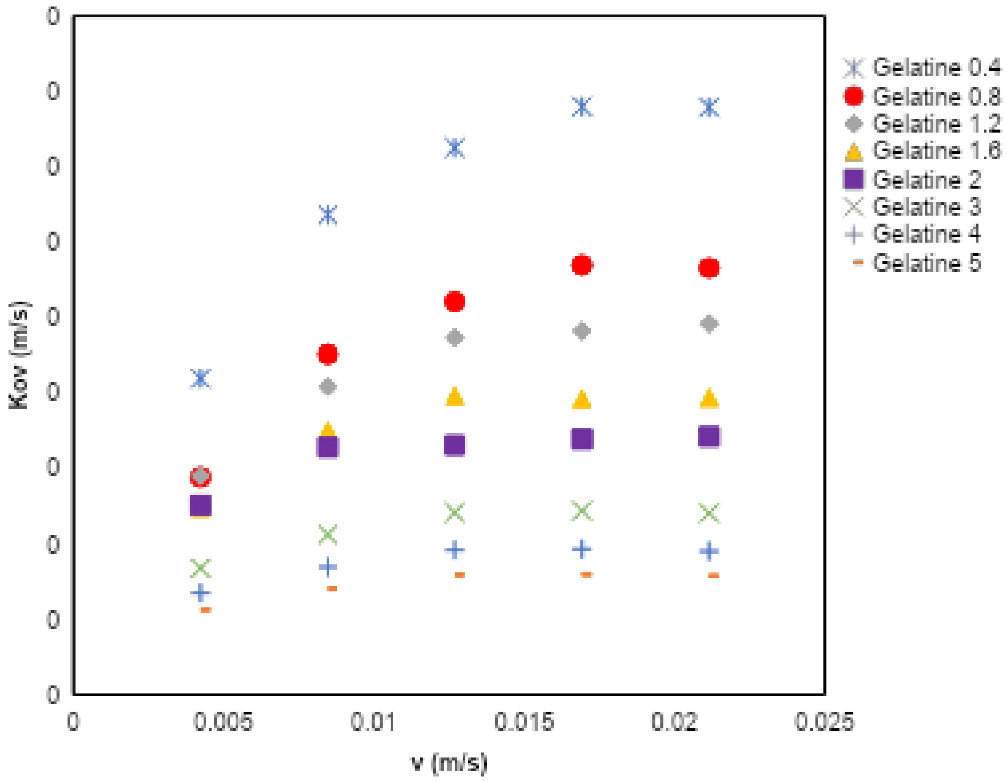

Figure S1. Overall mass transfer rate for experiments using clean water with increasing gelatine concentrations of 0.4-5 g/ L for increasing liquid velocities.
